# Severe Vaso-Occlusive Retinopathy in Systemic Lupus Erythematosus: A Case Series

**DOI:** 10.7759/cureus.13019

**Published:** 2021-01-30

**Authors:** Kshitiz Kumar, Shouvick Dan, Tushar K Sinha, Debashish Bhattacharya

**Affiliations:** 1 Vitreo-Retina, Disha Eye Hospital, Kolkata, IND

**Keywords:** systemic lupus erythematosus, brao, crvo, vaso-occlusive retinopathy, ranibizumab

## Abstract

This study aims to describe the clinical profile of severe vaso-occlusive retinal disorders in patients with systemic lupus erythematosus (SLE) and it is a retrospective case series. The clinical characteristics of three patients with SLE with vascular occlusions in four eyes were described. Branch retinal artery occlusion (BRAO) was present in all three patients with combined non-ischemic central retinal vein occlusion (NICRVO) in one patient and evolving ischemic CRVO in another patient. Additional branch retinal artery insufficiency was observed in the other eye of a patient with BRAO. Antinuclear antibody (ANA) titer was elevated in all patients. One patient had a positive lupus anticoagulant with elevated activated partial thromboplastin time (aPTT), and concurrent homocysteinemia was present in another patient. Intravitreal anti-vascular endothelial growth factor (ranibizumab) injection was administered to two eyes. Intravenous methyl prednisolone (IVMP) injection along with oral azathioprine was used in all patients with the need for anticoagulation in two patients along with SLE treatment. Vision in two eyes did not improve to the functional level despite aggressive therapy. Visually blinding severe vaso-occlusive retinopathy in the form of BRAO with or without CRVO can manifest in patients with SLE. Undetected antiphospholipid syndrome and homocysteinemia may be associated risk factors for such ophthalmic complications.

## Introduction

The systemic lupus erythematosus (SLE) is an autoimmune disorder affecting multiple organs due to underlying pathologic damage caused by tissue binding autoantibodies and immune complexes [1]. The frequency of ocular involvement in SLE is approximately 15%, and it is occasionally the presenting feature of the disease [2]. Retinal disorder in SLE is described as lupus-associated retinopathy, which is often an indicator of the severity of underlying systemic inflammation. The proportion of patients with retinopathy in SLE ranges from 3% in well-controlled to 29% in patients with the more active systemic disease [3]. Notably, the common manifestations of retinopathy are cotton wool spots, retinal hemorrhages, and optic disc edema caused by chronic occlusive changes in small retinal arterioles [4]. Severe vaso-occlusive retinopathy in the combination form of branch retinal artery occlusion (BRAO)/central retinal artery occlusion (CRAO) with central retinal vein occlusion (CRVO) is rare, with only a few reported cases in the literature [5-7].

In this series, we present the profile of three cases of severe vaso-occlusive retinopathy in known SLE patients describing their clinical characteristics and the involved treatment strategies.

## Case presentation

The diagnosis of SLE was reconfirmed based on the proper constellation of clinical findings and laboratory evidence per the European League Against Rheumatism (EULAR) and the American College of Rheumatology (ACR) criteria for the classification of SLE (Table 1). The EULAR/ACR classification requires an antinuclear antibody (ANA) titer of at least 1:80 on HEp-2 cells or an equivalent positive test at least once; otherwise, the patient is considered not to have SLE. If it is present, 22 'additive weighted' classification criteria are considered, comprising seven clinical domains (constitutional, hematological, neuropsychiatric, mucocutaneous, serosal, musculoskeletal, and renal) and three immunologic domains (antiphospholipid antibodies, complement proteins, and SLE-specific antibodies). Each criterion is assigned points, ranging from 2 to 10. Patients with at least one clinical criterion and 10 or more points are classified as having SLE [8]. All three patients had an additive score of greater than 22 individually.

**Table 1:** Clinical and Laboratory Findings of Patients With Vaso-occlusive Retinopathy in SLE *ANA: antinuclear antibody (normal <1:160); **anti-double standard DNA (normal <1:10); complement (C3&C4, normal: 16-38 mg/dl); CRP (normal <0.5); ESR (normal upto 20); aPTT (normal: 25-36 seconds); homocysteine (normal upto 12.2mmol/L). BRAO: branch retinal artery occlusion, CRVO: central retinal vein occlusion, NICRVO: non-ischemic central retinal vein occlusion, CRP: C-reactive protein, ESR: erythrocyte sedimentation rate, aPTT: activated partial thromboplastin time, SLE: systemic lupus erythematosus.

Patient	Age	Gender	ANA^* ^titer	Anti-dsDNA** titer	C3 and C4 level	Elevated CRP	Elevated ESR	Positive clinical history	Other laboratory tests
1. B/L BRA insufficiency	44	F	1:860	1:44	Low for both	+	+	Hemolytic anemia, leucopenia, non-erosive arthritis, pleural effusion, lupus nephritis, Malar rash	
2. BRAO with evolving CRVO	51	F	1:640	1:15	Low for both	+	+	Non-erosive arthritis, anemia, thrombocytopenia, Malar rash, fever	Lupus anti-coagulant +ve, aPTT (55 seconds)
3. BRAO with NICRVO	34	M	1:160	1:22	Low for both	+	+	Non-erosive arthritis, Lupus nephritis, hemolytic anemia	Homocysteinemia (21.6 mmol/L)

Case 1: Bilateral branch retinal arterial insufficiency with multiple BRAO

**Figure 1:**
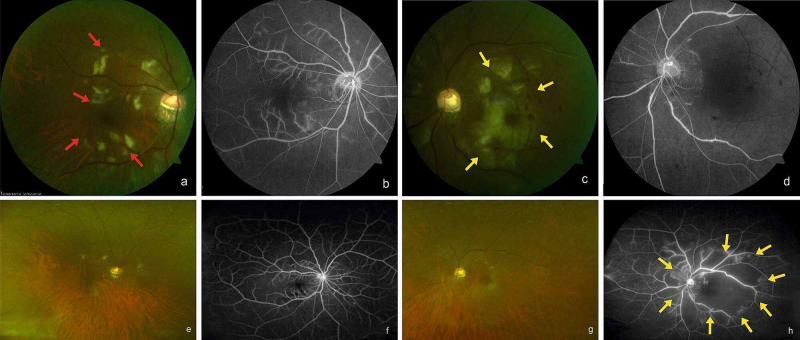
Case 1 Colour Fundus and Angiographic Images Fundus and angiographic images of the posterior pole of both eyes showing multiple aretriolitis (red arrows) in the right eye (a,b) and multiple BRAO (yellow arrows) in the left eye (e,f) at presentation. UWFI and UWFA images at six months showing resolving arteriolitis in the right eye (c,d) and large CNP areas (yellow arrows) with vascular staining in the left eye (d,h).

**Figure 2:**
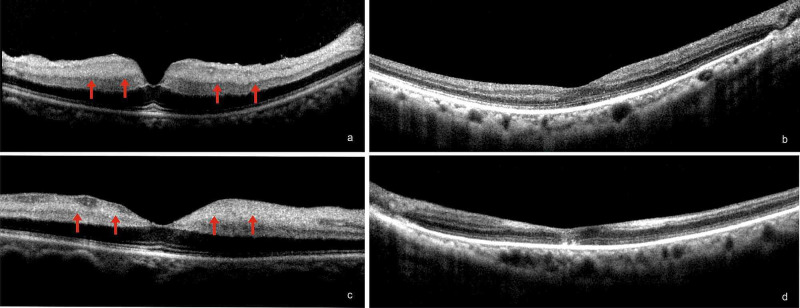
Case 1 OCT Images SD-OCT images: (a,c) at presentation showing hyperreflectivity of inner retinal layers (red arrows) suggestive of arteriolar occlusion in both eyes and (b,d) at six months showing normal retinal layers in the right eye and foveal thinning with disrupted outer retinal layers in the left eye.

A 44-year-old female with known SLE for ten years presented with the blurring of vision in both eyes of one-month duration. The best-corrected visual acuity (BCVA) was 20/200 in the right eye (RE) and 10/200 in the left eye (LE). Fundus evaluation revealed diffuse arteriolar narrowing, dot-blot hemorrhages, cotton wool spots in RE, sclerosed super-temporal artery with cattle-tracking of blood in multiple arterioles at the posterior pole, diffuse cotton wool spots, and retinal pallor corresponding to multiple branch retinal arteriolar occlusions in the LE (Figures 1a and 1c). Fluorescein angiography in both eyes demonstrated an inflammatory vasculopathy of retinal capillaries with associated multiple arteriolar affections in the early phase with pruned vascular appearance, arteriolar leakage in the late phase, capillary drop-outs, capillary non-perfusion areas (CNP), and non-filling of temporal retinal arterioles in LE (Figures 1b and 1d). Spectral-domain optical coherence tomography (SD-OCT) through the macula showed inner retinal layer hyperreflectivity with thickening and corresponding decreased reflectivity of outer retinal layers suggestive of arterial occlusion more marked in LE (Figures 2a and 2c). The central foveal thickness (CFT) was 209 µm in RE and 218 µm in LE. A diagnosis of LE multiple BRAO and RE branch retinal arteriolar insufficiencies was made. The patient was treated by a rheumatologist with three doses of intravenous methyl prednisolone (IVMP) 1 g per day, two cycles of intravenous rituximab 1 g at two-week intervals [9], followed by oral prednisolone in a tapering fashion, and a maintenance dose of hydroxychloroquine (HCQS) at 6.5 mg/kg/day [10] with azathioprine 50 mg daily. BCVA recovered to 20/40 in RE and 20/200 in LE within a week and was maintained at that level at the six-month follow-up with thinning of foveal layers in LE on OCT (Figures 2b and 2d). Ultra-wide field fundus angiography (UWFA) at six months showed multiple patchy CNP areas at the posterior pole in RE corresponding to affected arterioles and sclerosed temporal retinal arterioles with a large CNP area involving the macula and posterior pole (Figures 1e-1h). No neovascularisation elsewhere (NVE) was observed, and the patient was asked to remain under close observation.

Case 2: Unilateral BRAO with evolving CRVO (NICRVO ---> ICRVO)

**Figure 3:**
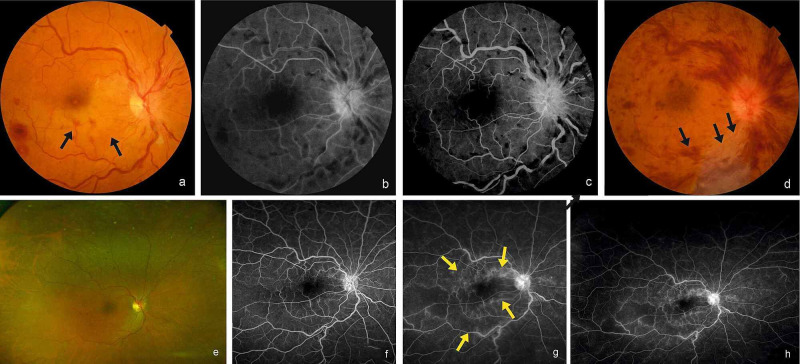
Case 2 Colour Fundus and Angiographic Images Fundus and angiographic images of the posterior pole of the right eye (a-c) at presentation showing inferotemporal BRAO (black arrows) with NICRVO. At six weeks fundus image (d) showing increased hemorrhages and macular edema and retinal pallor along infer-temporal artery (black arrows) suggestive of ischemic CRVO with BRAO in the right eye. At six months UWFI of the right eye (e) showing resolved hemorrhages, UWFA images of early phase (f) and late phases (g,h) showing vascular staining (yellow arrows) and minimal leakage with no NVE.

**Figure 4:**
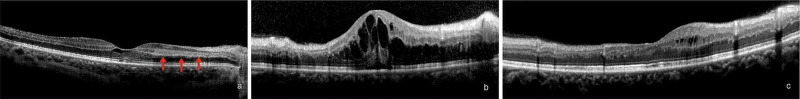
OCT Images of Case 2 SD-OCT images of the right eye: (a) at presentation showing hyperreflectivity of the inner retinal layer (red arrows) with a cystoid space; (b) at six weeks showing CME with NSD; (c) at six months showing resolved sub-foveal edema with parafoveal cystoid spaces.

A 51-year-old female with known SLE for 21 years presented with diminution of vision in RE for the past 15 days. She had RE BCVA 20/40. Fundus examination showed dot-blot hemorrhages, dilated tortuous veins, temporal peripapillary, and inferior macular retinal pallor with narrowing and cattle-tracking signs in the corresponding arterioles of RE (Figure 3a). On fundus fluorescein angiography (FFA), delayed filling of the branch retinal artery in the region of the pallor, delayed filling of veins with increased arteriovenous passage time of 28 s, and inflammatory vascular staining were observed (Figures 3b and 3c). SD-OCT through the macula revealed inner retinal layer hyperreflectivity with thickening and corresponding decreased reflectivity of the outer retinal layer suggestive of arterial occlusion in RE (Figure 4a). A diagnosis of combined infero-temporal BRAO with non-ischaemic CRVO (NICRVO) was made. Three loading doses of IVMP 1 g per day, subcutaneous low-molecular-weight heparin (enoxaparin, 1 mg/kg twice daily) for seven days followed by oral prednisolone in a tapering fashion and maintenance dose of HCQS at 6.5 mg/kg/day with azathioprine 50 mg daily and a regular dose of warfarin with an international normalized ratio (INR) goal of 2-2.5 was initiated by a rheumatologist. Patient BCVA was stable at one week with resolving retinal edema secondary to BRAO. However, the patient returned after six weeks with a reduction in RE BCVA to 20/200, fundus exhibiting diffusely increased flame-shaped hemorrhages, vascular tortuosity, macular edema, disc hyperemia with edema, and retinal pallor along the inferior branch retinal artery (Figure 3d), SD-OCT demonstrated cystoid macular edema (CME), neurosensory detachment (NSD), and CFT of 623 mm (Figure 4b). The current picture was suggestive of ITBRAO with ischemic CRVO (ICRVO). Following three loading doses of intravitreal ranibizumab 0.5 mg at monthly intervals, patient BCVA improved to 20/60, and CFT decreased to 248 mm (Figure 4c). UWFA performed at six months post-acute event revealed few patchy CNP areas inferiorly and temporally with no NVE (Figures 3e-3h). The patient was asked to review regularly.

Case 3: Unilateral BRAO with NICRVO

**Figure 5:**
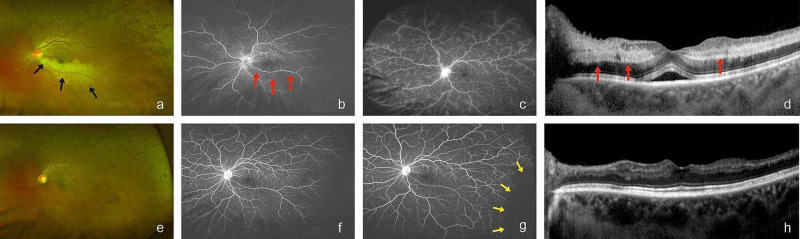
Case 3 Colour Fundus, Angiographic and OCT Images UWFI of the right eye (a) and UWFA images (b,c) at presentation showing BRAO (black and red arrows) with NICRVO. SD-OCT images of the right eye (d) at presentation showing inner-retinal layer hyperreflectivity (red arrows) and NSD. (e-h) UWFI, UWFA, and SD-OCT images at six months showing resolved hemorrhages, peripheral CNP areas (yellow arrows), and resolved macular edema.

A known male SLE patient (for four years) of 34 years age presented with acute loss of vision in LE of two days duration. LE BCVA was the perception of light (PL) only. Fundus evaluation in LE showed dot-blot hemorrhages, inferotemporal pallor along with narrowing and cattle-tracking of blood in the branch retinal artery, dilated tortuous veins, and disc hyperemia with edema (Figure 5a). FFA showed inflammatory vasculopathy, slow filling of the infero-temporal branch retinal artery, increased arteriovenous passage time of 23 seconds, and diffuse vascular staining (Figures 5b and 5c). SD-OCT was suggestive of BRAO with CME, NSD, and CFT of 560 mm (Figure 5d). A diagnosis of LE ITBRAO with NICRVO was made. The patient received three doses of IVMP 1 g per day, subcutaneous injections of heparin (enoxaparin, 1 mg/kg twice daily for seven days) along with folic acid (for homocysteinemia) under rheumatologist guidance followed by tapering doses of oral prednisolone and maintenance doses of HCQS and azathioprine as in previous patients. The patient underwent three loading doses of intravitreal ranibizumab injection 0.5 mg at monthly intervals. Post-intervention BCVA did not improve beyond finger counting at 1 m, although OCT showed resolution of macular edema and inner retinal layer hyperreflectivity with CFT of 266 mm (Figure 5h). UWFA at six months showed sheathed infero-temporal veins and arteries in the peripapillary area and CNP areas in the temporal retinal periphery beyond the equator with no NVE (Figures 5e-5g). The patient was asked to review at regular intervals.

 

## Discussion

We describe a well-documented series of severe vaso-occlusive retinopathy with variable features in SLE. Each patient had different combinations of vascular occlusions, and the likely pathogenesis is discussed separately.

CRAO/BRAO as the sole presentation of retinopathy in SLE is extremely rare. Bilateral CRAO has been reported twice and bilateral BRAO in SLE only once before [2,11,12]. The underlying pathogenesis of severe vaso-occlusive retinopathy is classically believed to be microangiopathy with diffuse capillary nonperfusion and small arterial or arteriolar occlusion in the retina characterized by microthrombosis and immune complex-mediated vasculopathy rather than true vasculitis [13]. Histologic examinations have described fibrinoid changes with thrombosis in vessel walls without evidence of inflammation [4]. As in the first patient with bilateral branch retinal insufficiency with multiple segmental BRAO in one eye, active vasculitis or evidence of emboli was not observed. Timely intervention not only helped improve vision in eyes with frank BRAOs but also saved the eye with arteriolar insufficiency from developing full-fledged occlusion. In addition to routine immunosuppressive treatment for acute ophthalmic presentation, rituximab was also used in this case. Rituximab selectively targets CD20+ B cells, leading to reduced T- and B-cell activation, prevention of antigen presentation, and a reduction in autoantibody production. The role of rituximab in lupus retinopathy has been described previously [9], and good outcomes were reported in an SLE patient with BRAO [12].

The second patient with BRAO with evolving CRVO from the non-ischaemic type into the ischaemic variant, despite being on an aggressive treatment protocol, makes an interesting case study. Only eight cases of different combinations of the branch or BRAO/CRAO and CRVO, as an ophthalmic emergency, have been reported in SLE [5-7,14]. The exact pathogenesis of simultaneous BRAO/CRAO with CRVO in SLE remains unknown, but the hypercoagulable state is considered to be an essential factor. It has been proposed that the presence of lupus anticoagulant and anticardiolipin antibodies in SLE patients adds to hypercoagulability, which is subsequently associated with an increased risk of developing severe vaso-occlusive retinopathy [13,15]. It was discussed in another study that patients with SLE and elevated antiphospholipid antibody levels are at higher risk for retinal vaso-occlusive disease [16]. Factors known to precipitate such a hypercoagulable state in these patients include acute infection, surgical and obstetric complications, trauma, neoplastic processes, and disease flare. In this patient, lupus anti-coagulant was positive with prolonged aPTT. However, the patient did not have any history of clinical features of the anti-phospholipid syndrome (APS), such as recurrent miscarriages at a younger age, deep vein thrombosis, or embolic phenomena, such as pulmonary embolism. In a cohort of patients with SLE, it was estimated that APS developed in 23% of the patients after 15 to 18 years of follow-up [17]. As noted in this case, the rare presentation of BRAO with evolving CRVO warrants a detailed systemic examination to exclude the emergence of APS in SLE and hence the need for regular screening of hypercoagulability.

The third patient with BRAO with NICRVO was similar to the case discussed above. However, the underlying hypercoagulability was probably accentuated by the raised levels of serum homocysteine. Increased serum homocysteine levels are seen in approximately 15% of patients with SLE and are associated with an increased risk of atherothrombotic events in this population [18]. Homocysteinemia was reported as a significant risk factor for retinal vascular occlusions alone [19]. Homocysteine is a highly reactive amino acid, and high levels of homocysteine are toxic to the vascular endothelium. The thrombotic effects of homocysteine have been described previously. Endothelial injury by the release of free radicals, creating an environment of hypercoagulability, and modification of the vessel wall is probably the key mechanism of thrombotic and atherosclerotic complications [19]. Correcting nutritional inadequacy of folic acid and vitamin B lowers homocysteine levels in most patients [20]. Despite correction of the increased homocysteine level, aggressive therapy with immunosuppression, and anticoagulation in this patient, the vision did not recover to the functional level. This is the first report of BRAO with CRVO in SLE with homocysteinemia.

## Conclusions

Through this series, we highlight atypical presentations of ‘lupus retinopathy’, such as BRAO and BRAO with CRVO (ischemic and non-ischemic type). Such severe vaso-occlusive retinopathies are visually blinding conditions that warrant thorough clinical and laboratory evaluations along with urgent intervention in the form of immunosuppression, anticoagulation, and treatment for underlying hypercoagulability. Rituximab may have a role in the setting of BRAO, but this possibility needs to be explored on a larger scale. Clinicians should have a high level of suspicion for undetected antiphospholipid syndrome in SLE patients presenting with CRVO with arteriolar occlusion. Underlying hypercoagulability due to homocysteinemia might be present in SLE patients, resulting in severe vascular occlusion in the form of CRVO with BRAO. This case series will help clinicians, especially ophthalmologists, become increasingly aware of such ocular presentations, and their line of management in SLE patients.
